# Supplementary material for: Modulation of cannabinoid receptor 2 alters neuroinflammation and reduces formation of alpha-synuclein aggregates in a rat model of nigral synucleinopathy
Source: J Neuroinflammation. 2024 Sep 27;21:240. doi: 10.1186/s12974-024-03221-5 (PMC11438102; doi:10.1186/s12974-024-03221-5)
Supplement: Supplementary file 1 — Supplementary Material 1 [file 12974_2024_3221_MOESM1_ESM.pdf]

Table S1. Myeloid PBMC flow panel

Antibody	Manufacturer	Product number	Dilution
anti-Rat CD45-BUV395	BD Biosciences	740258<br>RRID:AB_2740002	1:50
anti-Rat His48-FITC	Invitrogen	11-0570-82<br>RRID:AB_465100	1:200
Anti-Rat CD43-PE-Vio770	Miltenyi	130-107-721<br>RRID:AB_2658086	1:100
Anti-rat CD3-Viogreen	Miltenyi	130-103-126<br>RRID:AB_2657100	1:200
Anti-rat CD4-APC-Vio770	Miltenyi	130-107-504<br>RRID:AB_2657949	1:100
Anti-ms/rat MHCII-PerCP-Vio700	Miltenyi	130-107-877<br>RRID:AB_2652898	1:100
Anti-rat CD11b-Pac Blue	BioRad	MCA275PB<br>RRID:AB_566459	1:50
Anti-rat RP1-BV786	BD Biosciences	743058<br>RRID:AB_2741253	1:20
Anti-rat CD172a-APC	R&D systems	FAB7307A	1:100
Anti-rat CD32	BD Biosciences	550271<br>RRID:AB_393568	1:100

Table S2. Lymphoid PBMC flow panel

Antibody	Manufacturer	Product number	Dilution
Anti-rat CD45RA-PECy7	Biolegend	202315<br>RRID:AB_2565942	1:200
Anti-rat CD3-Viogreen	Miltenyi	130-103-126<br>RRID:AB_2657100	1:200
Anti-rat CD4-APC-Vio770	Miltenyi	130-107-504<br>RRID:AB_2657949	1:100
Anti-CD8a-PerCP-Vio700	Miltenyi	130-108-914<br>RRID:AB_2659483	1:100
Anti-rat CD25-BV786	BD Biosciences	742757<br>RRID:AB_2741022	1:50
Anti-ms/rat Foxp3-AF647 *stained following intracellular permeabilization	R&D systems	FAB7307A	1:50
Anti-rat CD32	BD Biosciences	550271<br>RRID:AB_393568	1:100

Table S3. Brain Immune cell flow panel

Antibody	Manufacturer	Product number	Dilution
anti-Rat CD44H-FITC	Miltenyi	130-107-854 RRID:AB_2658192	1:50
Anti-Rat CD62L-PE	BD Biosciences	551398 RRID:AB_394182	1:200
Anti-rat CD45RA-PECy7	Biolegend	202315 RRID:AB_2565942	1:200
Anti-rat CD3-Viogreen	Miltenyi	130-103-126 RRID:AB_2657100	1:200
Anti-rat CD4-APC-Vio770	Miltenyi	130-107-504 RRID:AB_2657949	1:100
Anti-CD8a-PerCP-Vio700	Miltenyi	130-108-914 RRID:AB_2659483	1:100
Anti-rat CD25-BV786	BD Biosciences	742757 RRID:AB_2741022	1:50
Anti-ms/rat Foxp3-AF647 *stained following intracellular permeabilization	R&D systems	FAB7307A	1:50
Anti-rat CD32	BD Biosciences	550271 RRID:AB_393568	1:100

Table S4. Western and IHC antibodies

Antibody	Company	Product #	Loading protein concentration	Concentration
**Western antibodies**				
TH	Millipore	AB152 RRID:AB_390204	10ug	1:2000
Asyn (4B12)	BioLegend	807801 RRID:AB_2564730	10ug	1:500
DAT	Novus Bio	NBP2-22164	10ug	1:2000
IBA1	Abcam	AB5076 RRID:AB_2224402	10ug	1:1500
Phospho-TH	Phospho solutions	P1580-40 RRID:AB_2492279	10ug	1:1000
GFAP	Dako	Z0334 RRID:AB_10013382	15ug	1:2500
pSer129 (EP1536Y)	Abcam	AB51253 RRID:AB_869973	15ug	1:500
**IHC antibodies**				
IBA1	Wako	019-19741 RRID:AB_839504		1:500
TH	Immunostar	22941 RRID:AB_572268		1:1000
Asyn (4B12)	BioLegend	807801 RRID:AB_2564730		1:2500
pSer129 (EP1536Y)	Abcam	AB51253 RRID:AB_869973		1:2500
CD68	Biorad	MCA341R RRID:AB_2291300		1:2000
MHCII	Biorad	MCA46GA RRID:AB_567369		1:1000
CD163	Biorad	MCA342R RRID:AB_2074557		1:1000

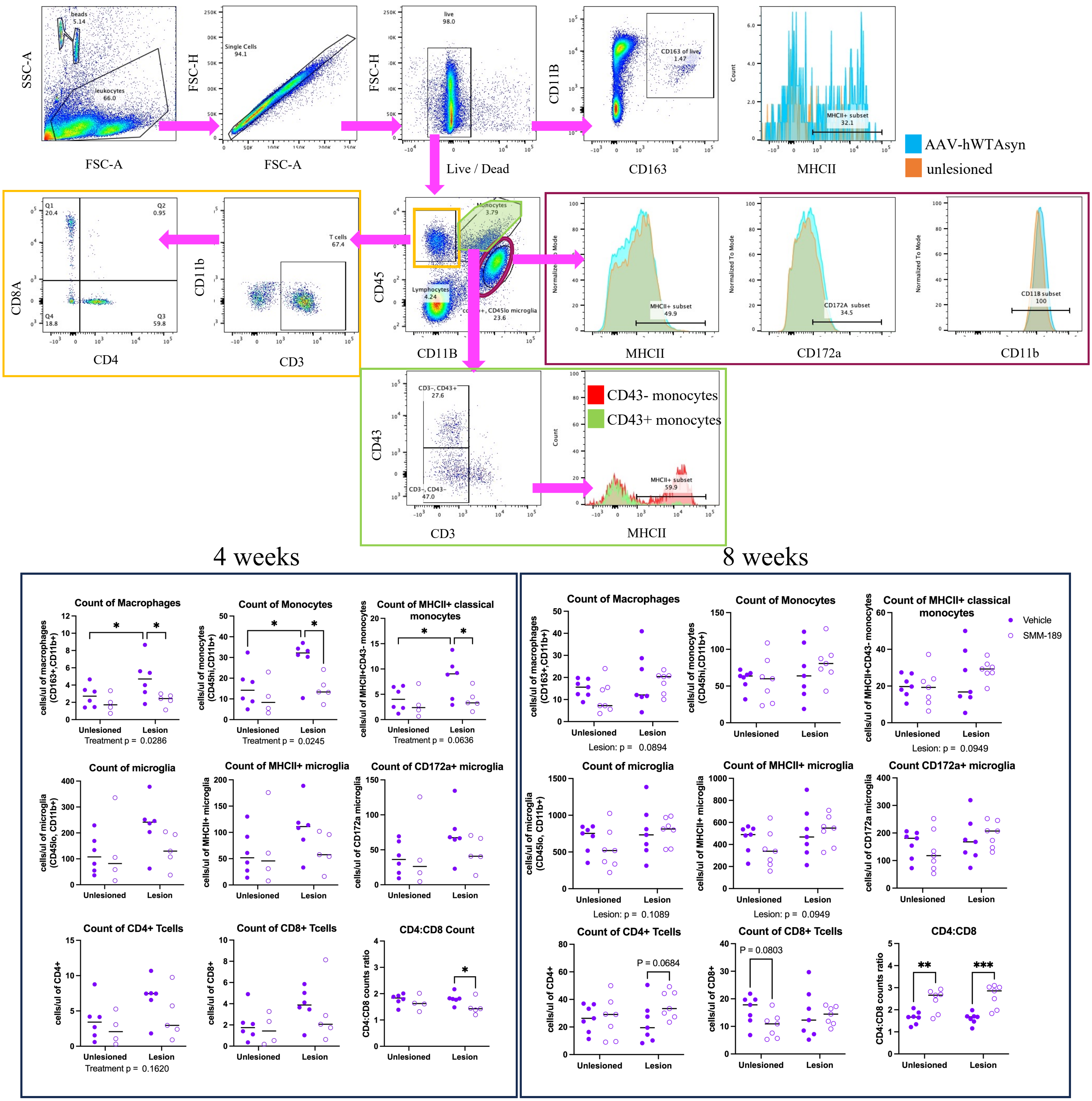

Figure S1. Rat brain immune cells flow gating strategy and cell counts. Cells were initially gated by SSC and FSC, doublets excluded and live cells selected as those that did not take up live/dead fixable stain. Live cells were further gated by CD45 and CD11b where microglia (CD45lo, CD11b+), lymphocyte (CD45+CD11b-), and monocytes (CD45hi, CD11b+) populations were identified. Microglia subsets were further defined by MHCII, CD172a and CD11b frequencies and MFI. Monocytes were gated on CD3-, and two populations of monocytes were identified by CD43lo (classical monocytes) and CD43hi (non-classical monocytes) and monocyte activation determined by frequencies and MFI of MHCII. Live cells were also gated for CD163+ macrophages and further evaluated for frequency and MFI of MHCII. Lymphocytes were gated as CD3+CD11b- and further defined by CD4 and CD8 expression. Quantification of immune cell counts in the brain reflect similar SMM-189 induced significant frequency reductions in monocyte and macrophage at 4 weeks, and similar rebalancing of myeloid counts between treatments at 8 weeks.

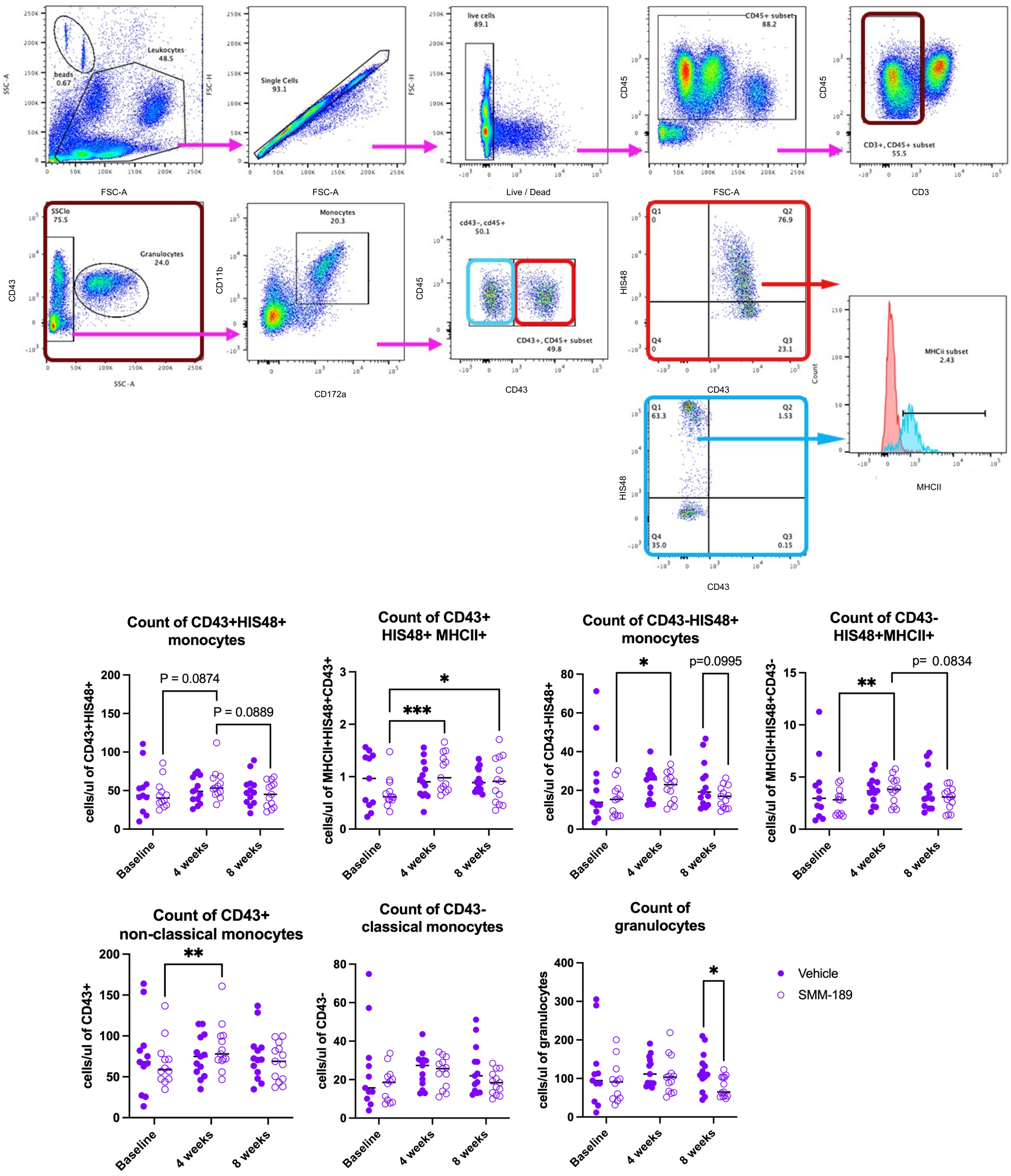

Figure S2. Rat PBMC myeloid cells flow gating strategy and cell counts. Cells were initially identified by SSC and FSC, doublets excluded and live cells selected as those that did not take up live/dead fixable stain. Immune cells were gated as CD45+ and monocytes further defined by CD3-. Granulocytes were excluded by size with high SSC, and monocytes further defined by CD11b+/CD172a+. Two populations of monocytes were initially identified by CD43 and further defined by His48 expression: classical monocytes (CD43lo/HIS48+) and non-classical monocytes (CD43hi/His48lo) and monocyte activation determined by MHCII MFI. Quantification of PBMC immune cell counts at baseline, 4 and 8 weeks after AAV overexpression of Asyn reflect similar SMM-189 induced frequency increases in non-classical CD43+ and decreases in classical CD43- monocyte populations.

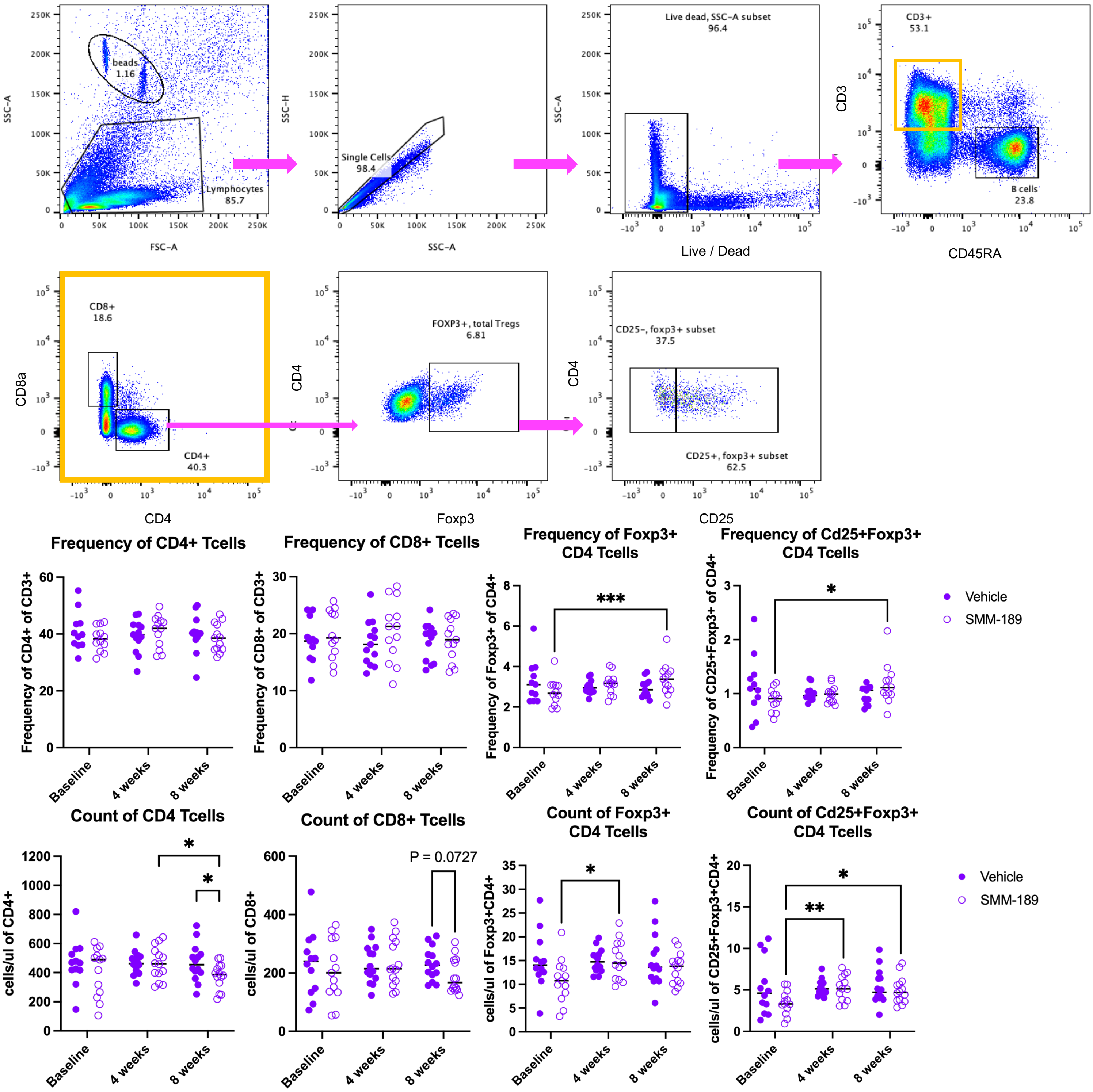

Figure S3. Flow gating strategy for rat lymphocytes and elevation of peripheral Tregs in SMM-189-treated rats (high cohort) after 7 weeks of treatment. Cells were initially identified by SSC and FSC, doublets excluded and live cells selected as those that did not take up live/dead fixable stain. Lymphocytes were gated as CD3+ and subpopulations of T cells defined by CD8 or CD4. Tregs were also identified from CD4 populations (foxp3+ CD4+) with subpopulations separated by CD25 expression. B cells were identified as CD3-CD45RA+ populations. PBMCs were evaluated by flow cytometry at baseline, 4 and 8 weeks after AAV2/5-hAsyn from high cohort rats. Quantification of lymphocyte population frequencies and counts (cells/ul) show a significant increase in the frequency of CD4+ Tregs (Foxp3+ and Foxp3+/CD25+) across time in SMM-189 treated rats. Frequncy analysis finds no differences in T cell CD4+ or CD8+ populations between treatments at any timepoints, yet count analysis finds both CD4+ and CD8+ Tcells reduced at 8 weeks in SMM-189-treated rats compared to vehicle-treated.

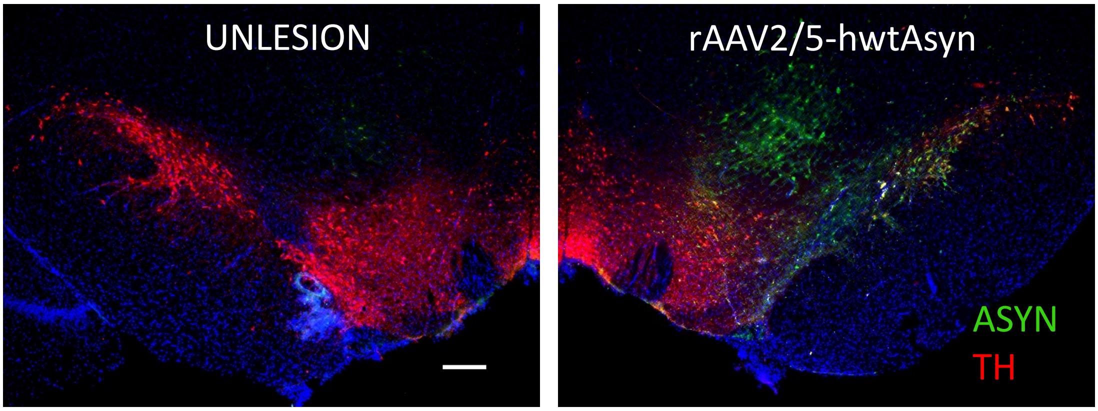

Figure S4. Targeting of rAAV2/5-hwtAsyn in the rat substantia nigra.
Representative immunofluorescent staining of tyrosine hydroxylase (TH; red) and Asyn (green) with DAPI (blue) demonstrating unilateral presence of Asyn in the TH+ nigra neurons. Scale bar 300µm.

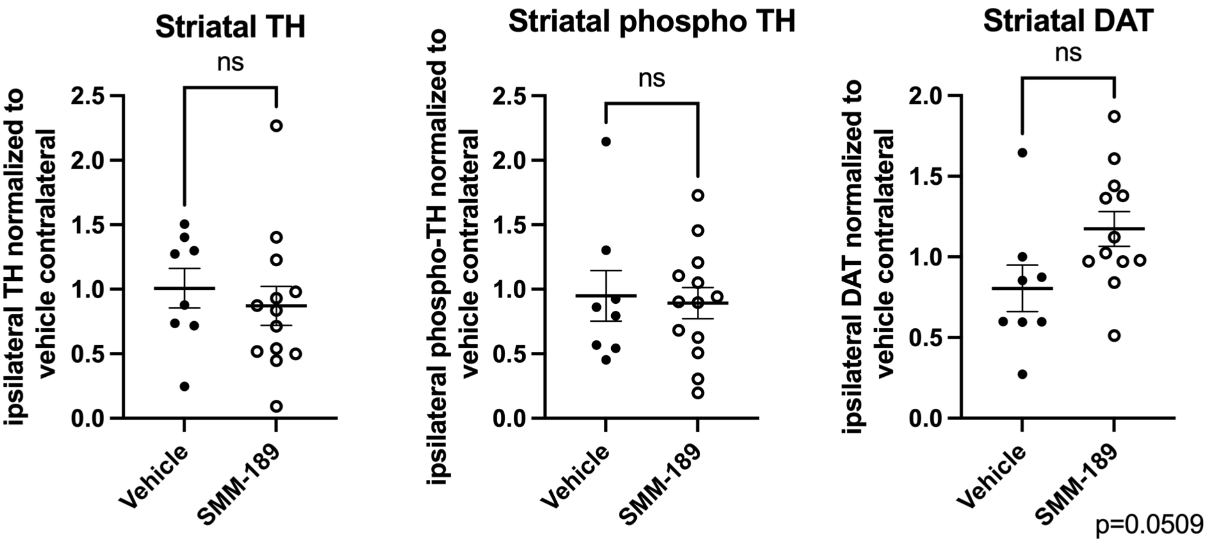

Figure S5. Protein markers for dopaminergic neurons in striatum do not change by CB2 modulation with SMM-189. Striatal TH, phosphoTH and dopamine transporter (DAT) protein expression normalized to its own total protein and to the contralateral hemisphere of vehicle-treated rats show no differences between treatments with a strong trend to increased DAT in SMM-189-treated rat ipsilateral striatum.

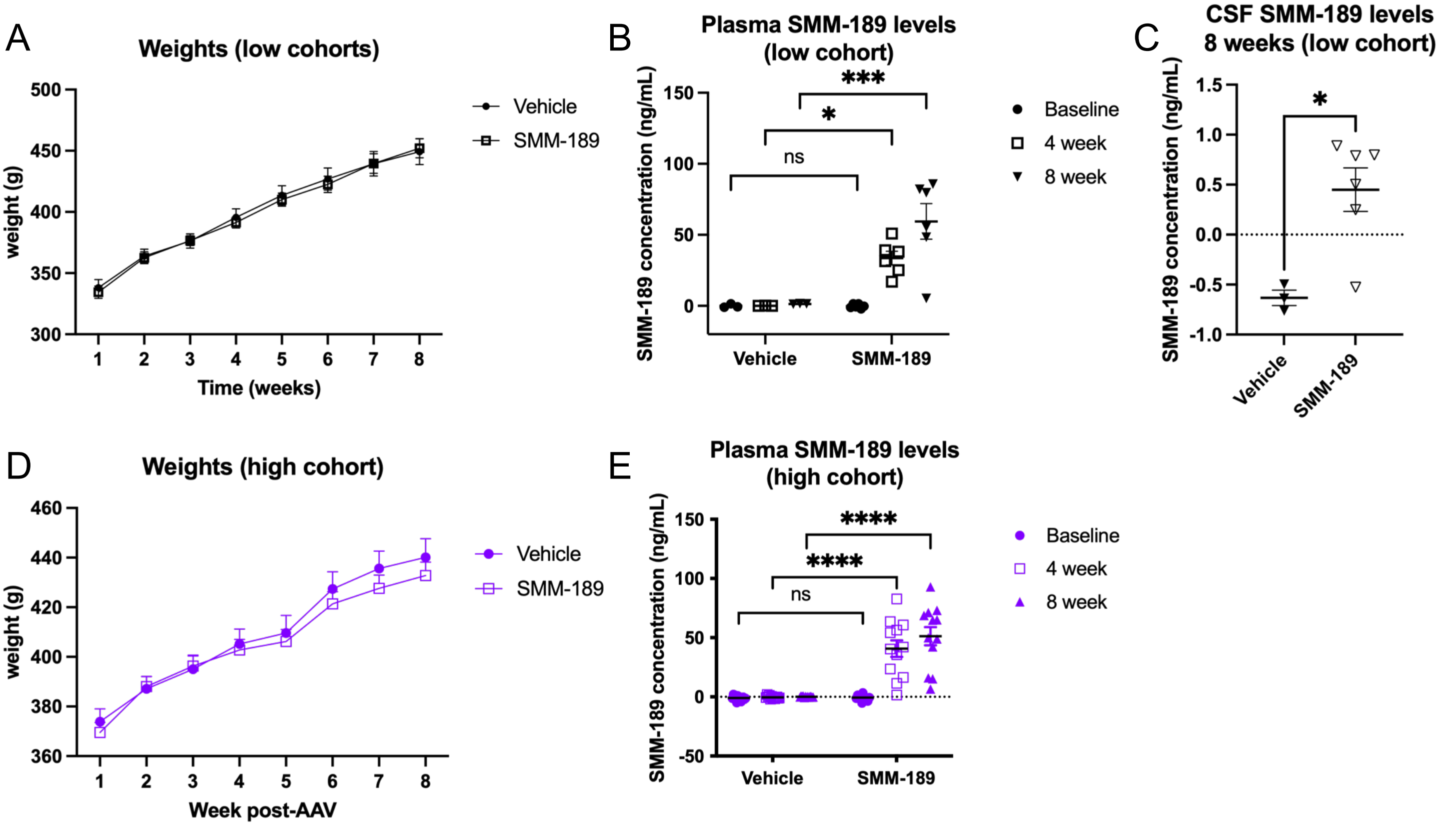

Figure S6. Animal body weights and levels of SMM-189 in plasma and CSF. A,D) Weekly body weights did not demonstrate differences between treatment groups over 8 weeks for either low or high cohort. B,E) Blood was collected from the tail vein at baseline and every 4 weeks post-AAV. Blood was collected from tail vein approximately 2 hours after SMM-189 or vehicle dose. Significant increases were seen in the level of plasma SMM-189 in animals receiving SMM-189 treatment at both 4 and 8 weeks in SMM-189 treated rats compared to vehicle treatment. C) CSF was collected at endpoint and ~2 hours after peripheral treatment of either vehicle or SMM-189 in a subset of low cohort animals and found significantly elevated in SMM-189-treated rats.

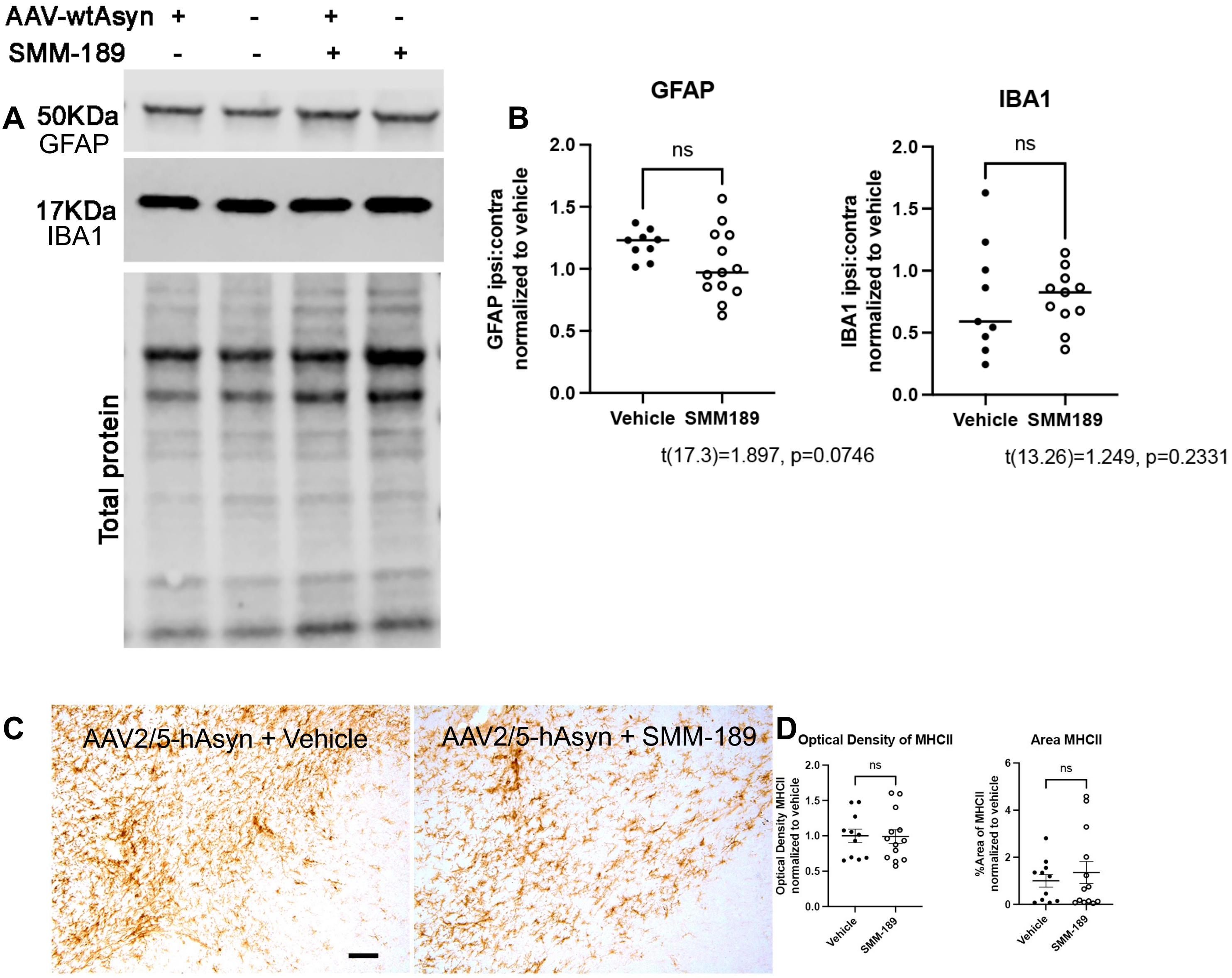

Figure S7. Protein markers for astrocytes or microglia in the striatum and MHCII immunostaining does not change by CB2 modulation with SMM-189. A) Representative blots for GFAP (50KDa) and IBA1 (17KDa) of striatal lysates from ipsilateral and contralateral hemispheres of low cohort rats. B) Quantification of GFAP and IBA1 relative expression normalized to its own total protein and as a ratio of ipsilateral to contralateral levels show no difference between treatment groups. C) Representative microphotographs of MHCII expression in the lesioned nigra of rats treated with either vehicle or SMM-189. D) Quantification of nigral MHCII-ir reveal no difference in the intensity or area between treatments.

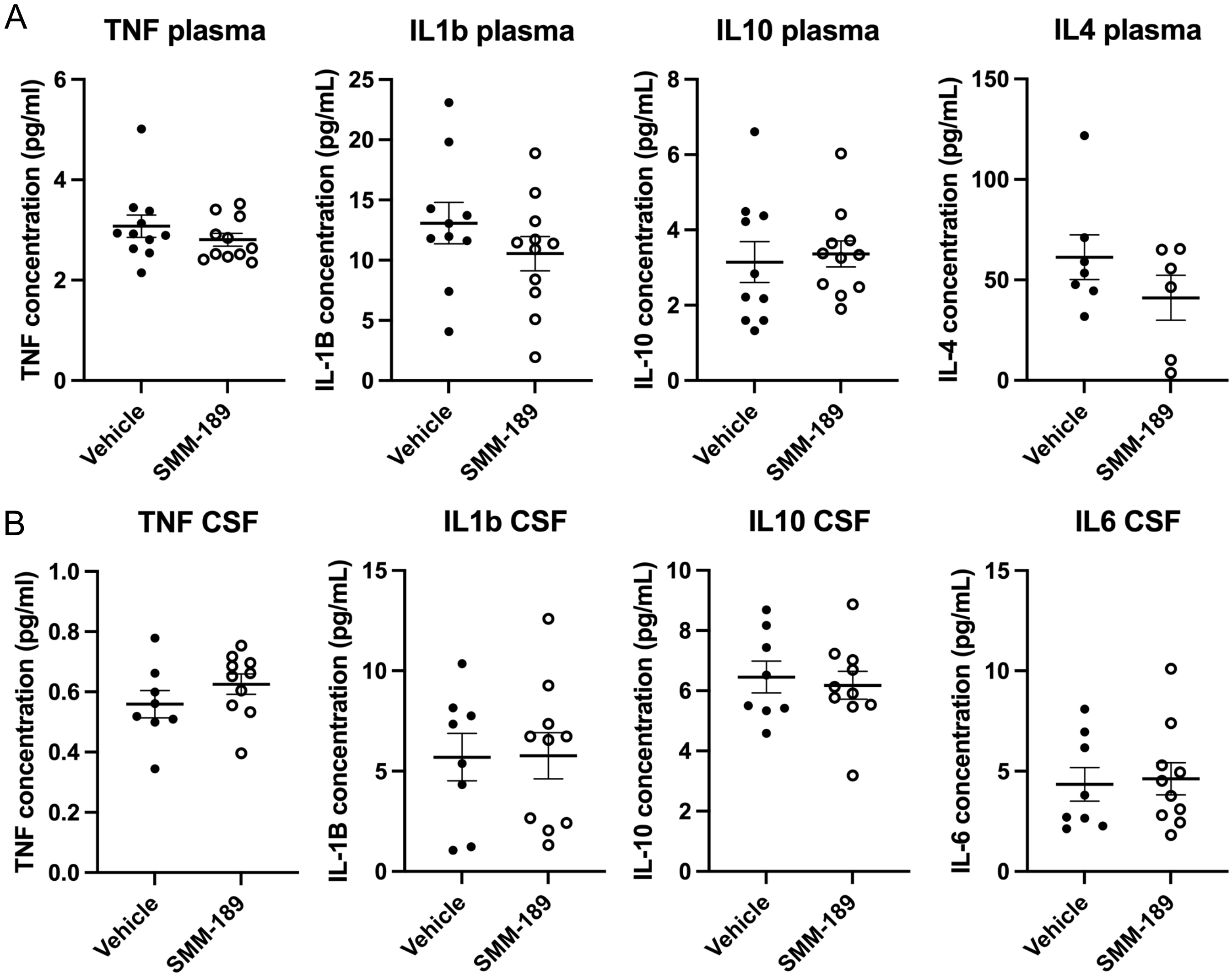

Figure S8. Cytokine levels in biofluids of SMM-189- and vehicle-treated rats at 8 weeks. Prior to sacrifice, blood and CSF were collected and for cytokine analysis using . A) No differences were found in plasma TNF, IL-1b, IL-10 or IL-4 between treatments. B) No differences were found in CSF TNF, IL-1b, IL-10 or IL-6 between treatments.

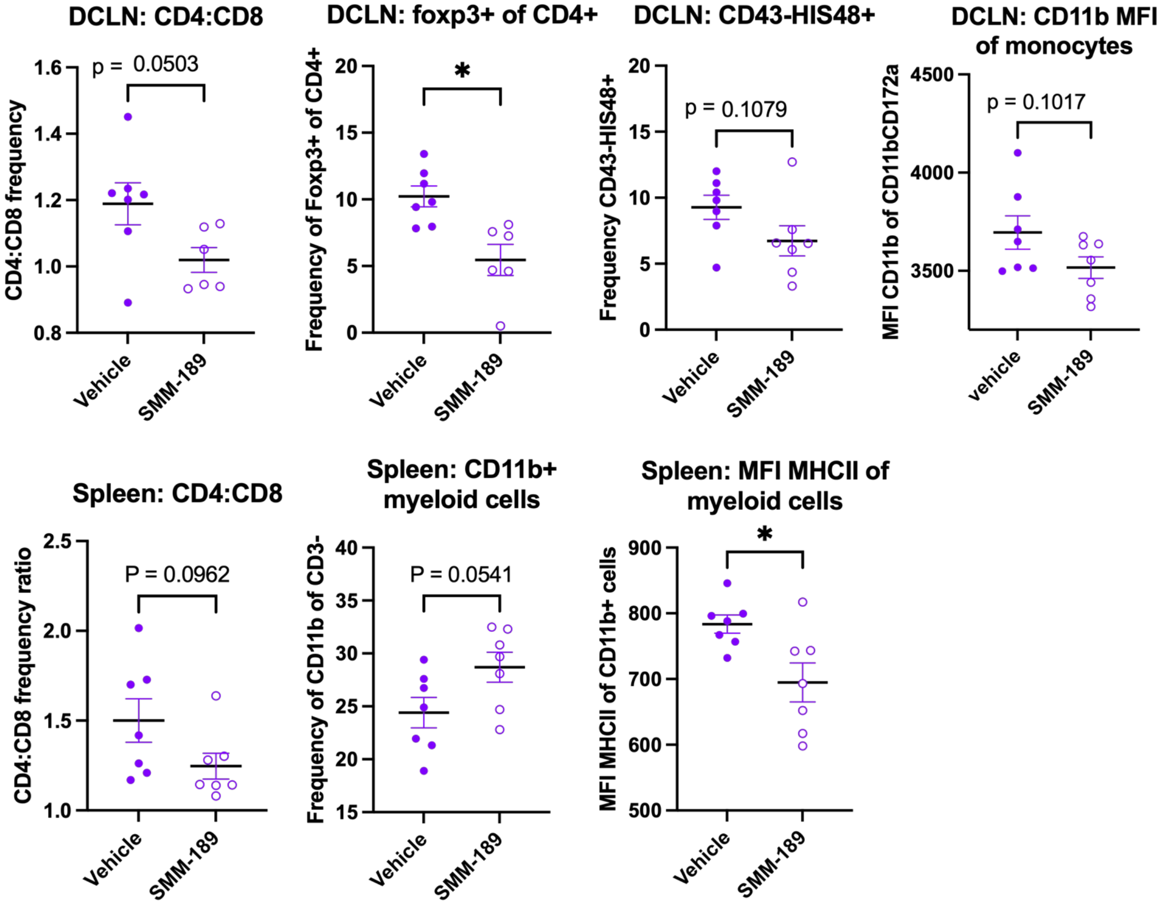

Figure S9. Immune cell populations in deep-cervical lymph nodes (DCLN) and splenocytes are minimally affected in SMM-189-treated rats (high cohort) after 7 weeks of dosing. Quantification of immune cell frequencies in the DCLN reflect a trend to reduced CD4 to CD8 ratio (p=0.0503) and significant reduction in Tregs (foxp3+CD4+) from SMM-189 peripheral treatment. No frequency difference were seen in DCLN myeloid populations including classical CD43-His48+ or CD11b+ monocytes. Quantification of immune cell frequencies in splenocytes identify no effect of treatment on CD4 to CD8 lymphocyte ratio, but myeloid populations (CD11b+) demonstrate a trend to increase and significant reductions in the mean fluorescent intensity (MFI) of MHCII+CD11b+ myeloid cells due to SMM-189 treatment.
